# *Sphingomonas* *paucimobilis* Bloodstream Infections Associated with Contaminated Intravenous Fentanyl^1^

**DOI:** 10.3201/eid1501.081054

**Published:** 2009-01

**Authors:** Lisa L. Maragakis, Romanee Chaiwarith, Arjun Srinivasan, Francesca J. Torriani, Edina Avdic, Andrew Lee, Tracy R. Ross, Karen C. Carroll, Trish M. Perl

**Affiliations:** Johns Hopkins University School of Medicine, Baltimore, Maryland, USA (L.L. Maragakis, A. Lee, K.C. Carroll, T.M. Perl); Johns Hopkins University Bloomberg School of Public Health, Baltimore (R. Chaiwarith); Centers for Disease Control and Prevention, Atlanta, Georgia, USA (A. Srinivasan); University of California, San Diego, California, USA (F.J. Torriani); The Johns Hopkins Hospital, Baltimore (E. Avdic, T.R. Ross); 2 These authors contributed equally to this article.

**Keywords:** Sphingomonas, outbreak, healthcare-associated infections, compounding pharmacy, research

## Abstract

Nationally distributed medications from compounding pharmacies, which typically adhere to less stringent quality-control standards than pharmaceutical manufacturers, can lead to multistate outbreaks. We investigated a cluster of 6 patients in a Maryland hospital who had *Sphingomonas paucimobilis* bloodstream infections in November 2007. Of the 6 case-patients, 5 (83%) had received intravenous fentanyl within 48 hours before bacteremia developed. Cultures of unopened samples of fentanyl grew *S. paucimobilis;* the pulsed-field gel electrophoresis pattern was indistinguishable from that of the isolates of 5 case-patients. The contaminated fentanyl lot had been prepared at a compounding pharmacy and distributed to 4 states. Subsequently, in California, *S. paucimobilis* bacteremia was diagnosed for 2 patients who had received intravenous fentanyl from the same compounding pharmacy. These pharmacies should adopt more stringent quality-control measures, including prerelease product testing, when compounding and distributing large quantities of sterile preparations.

## Abstract 2

Compounding pharmacies should be required to follow good manufacturing practices, including end-product sterility testing.

Compounding pharmacies are licensed or registered by state pharmacy boards to combine “reasonable quantities” of ingredients to fill a valid prescription from a licensed practitioner for an individual patient (*1*). Some pharmacies, however, have moved beyond this role and, in anticipation of receiving routine orders, prepare larger quantities of compounded preparations for national distribution to healthcare facilities (*2*,*3*). Nationally distributed medications from compounding pharmacies, which typically adhere to less stringent quality-control standards than pharmaceutical manufacturers, can lead to multistate outbreaks that may be difficult to detect. Sunenshine et al. recently reported a 5-state outbreak of *Serratia marcescens* bloodstream infections associated with contaminated intravenous magnesium sulfate from a compounding pharmacy (*3*). Other reported outbreaks caused by contaminated medications from compounding pharmacies include the following: *S. marcescens* infections associated with betamethasone injection, *Pseudomonas putida* septicemia caused by use of contaminated flush solutions in a special-care nursery, *Burkholderia cepacia* infections caused by contaminated intravenous flush solutions, *Pseudomonas fluorescens* bloodstream infections associated with a heparin/saline flush, and *Exophiala dermatitidis* infections caused by injection of contaminated steroids (*4–10*; Table 1).

**Table 1:** Recently published reports of infectious outbreaks associated with contaminated medications prepared at compounding pharmacies, United States, 2002–2007

Reference	Organism	Infection ( no. patients)	Mode of transmission	Location of outbreak
(*3*)	*Serratia marcescens*	Bloodstream infections (18)	Intravenous magnesium sulfate	California, New Jersey, North Carolina, New York, Massachusetts
(*4*)	*S. marcescens*	Meningitis, epidural abscess, or joint infection (11)*	Epidural or intra-articular injection of betamethasone	California
(*6*)	*Burkholderia cepacia*	Bloodstream infections and sepsis (2 pediatric patients)	Intravenous antibiotic-lock flush solution	Connecticut
(*7*)	Hepatitis C	Acute hepatitis C (16)	Injected radiopharmaceutical for myocardial perfusion study	3 clinics in Maryland
(*8*,*10*)	*Pseudomonas fluorescens*	Bloodstream infections (64)	Heparin/saline intravenous flush	Missouri, New York, Texas, Michigan, South Dakota
(*9*)	*Exophiala dermatitidis*	Meningitis (*5*)†	Epidural injection of methylprednisolone‡	2 pain management clinics in North Carolina

*3 case-patients died. †1 case-patient died. ‡Prepared by a compounding pharmacy in South Carolina and supplied to hospitals and clinics in 5 states.

*Sphingomonas paucimobilis* is an aerobic bacterium found in soil and water; it is a rare cause of healthcare-associated infections (*11*,*12*). *S. paucimobilis* has been reported to cause outbreaks of bacteremia among immunocompromised patients in hematology and oncology units; these outbreaks are possibly related to bacterial colonization of hospital water systems (*13*,*14*). An *S. paucimobilis* outbreak in mechanically ventilated neonates was linked to contaminated temperature probes (*15*). In November 2007, The Johns Hopkins Hospital Department of Hospital Epidemiology and Infection Control initiated an outbreak investigation after being notified by the hospital’s microbiology laboratory of the growth of *S. paucimobilis* in several patients’ blood cultures over a 2-week period.

## Methods

The Johns Hopkins Hospital is a 926-bed, tertiary-care, academic hospital in Baltimore, Maryland, USA. For this investigation, we defined a case-patient as any patient in our hospital whose cultures of blood or of other normally sterile body sites grew *S. paucimobilis* in November 2007. *S. paucimobilis* isolates were characterized as gram-negative rods that are yellow-pigmented, glucose nonfermenting, and weakly oxidase positive; they were preliminarily identified by the BD Phoenix Automated Microbiology System (BD Diagnostics, Inc. Sparks, MD, USA). All *S. paucimobilis* isolates were confirmed by cell wall fatty acid analysis using gas liquid chromatography (Sherlock Microbial Identification System version 4.5, library 5.0; MIDI, Inc. Newark, DE, USA). Microbiology records from January 2006 through November 2007 were examined to identify case-patients and to establish the baseline rate of *S. paucimobilis* bacteremia. We identified common exposures for case-patients and focused on intravenous infusions, medications, and contrast agents and on case-patients’ clinical signs, treatments, and outcomes. Because most of the blood cultures growing *S. paucimobilis* were collected in BacT/Alert FA bottles containing activated charcoal (bioMérieux, Durham, NC, USA), we cultured noninoculated bottles from clinical units by placing them directly into the blood culture instrument to assess for intrinsic contamination. On the basis of information from the medical record review, samples for bacterial culture were taken from 4 implicated lots of intravenous fentanyl mixed in 0.9% sodium chloride solution. All 4 lots came from an out-of-state compounding pharmacy, hereafter called pharmacy A. All *S. paucimobilis* isolates were strain typed by pulsed-field gel electrophoresis (PFGE) after digestion with *XbaI* using standard methods and interpreted according to criteria established by Tenover et al. (*16*).

We investigated the possibility that fentanyl had been tampered with or diverted by tracing the narcotic chain of custody, reviewing controlled substance procedures, visually inspecting fentanyl bags for signs of tampering, testing fentanyl concentrations, and analyzing personnel access records from the automated medication management system (Pyxis, Cardinal Health; www.cardinal.com/us/en/providers/products/pyxis/index.asp). The investigation was coordinated with public health authorities, including the Centers for Disease Control and Prevention (CDC) and the Baltimore City Health Department. CDC performed a multistate case-finding investigation by working with the compounding pharmacy to trace the distribution of implicated lots of intravenous fentanyl and by asking recipient healthcare institutions whether they had identified cases of *S. paucimobilis* bacteremia. We notified the US Food and Drug Administration (FDA) of our findings, and the FDA investigated compounding practices at pharmacy A. The Johns Hopkins University Institutional Review Board approved this study and waived informed consent.

## Results

**Figure 1:**
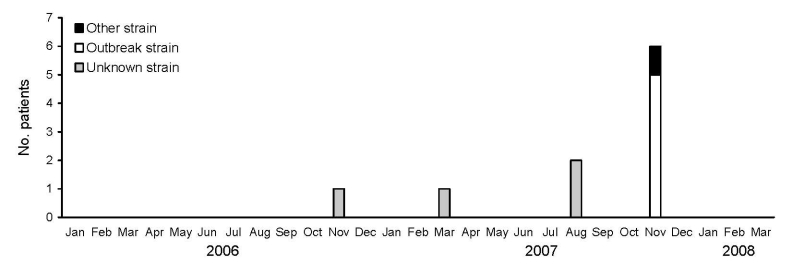
Epidemiologic curve showing the number of patients with *Sphingomonas paucimobilis* bacteremia at The Johns Hopkins Hospital, Baltimore, Maryland, USA, January 2006 through March 2008.

*S. paucimobilis* was isolated from the blood cultures of 6 patients; the samples were collected from November 11 through November 23, 2007. The organism was not isolated from cultures of any other body site. Case-patients had various underlying medical conditions and had been admitted to different hospital units: neurologic intensive care unit (3 patients), medical intensive care unit (1 patient), oncology center (1 patient), and medicine unit (1 patient) (Table 2). *S. paucimobilis* grew in multiple sets of blood cultures (5 patients) and from blood cultures collected on >1 date (3 patients). In the preceding 22 months, *S. paucimobilis* had been isolated from blood cultures of 4 patients (Figure 1). As a result of *S. paucimobilis* bloodstream infections, 5 of the case-patients reported here received antimicrobial drug treatment and had central intravenous catheters or implanted medication ports removed and replaced. One of these patients had an adverse reaction (rash and renal insufficiency) to antimicrobial drug treatment. All 5 patients treated with antimicrobial agents became free of *S. paucimobilis* bloodstream infection and survived. One patient (patient 5, Table 2) died of group A streptococcal sepsis before blood culture results for *S. paucimobilis* were available.

**Table 2:** Demographic and clinical characteristics of patients with *Sphingomonas paucimobilis* bloodstream infection, United States, 2007*

Patient no./ age, y/gender	Hospital unit (US state)	Clinical diagnosis	Date(s) of fentanyl administration†	Date(s) of infection	Treatment	Outcome	PFGE strain‡
1/65/M	Medicine (MD)	Osteomyelitis; MRSA wound infection	NA	Nov 14	ADT, central line removed and replaced	Survived	Unique
2/64/M	NCCU (MD)	Subarachnoid hemorrhage	Oct 29–31, Nov 2–3, Nov 11–22	Nov 14, Nov 18	ADT, central line removed and replaced	Survived	A
3/46/F	NCCU (MD)	Subarachnoid hemorrhage	Nov 10–11 Nov 16–18	Nov 11 Nov 17 Nov 19	ADT, central line removed and replaced	Survived	A
4/69/F	NCCU (MD)	Subarachnoid hemorrhage	Nov 18	Nov 18	ADT, central line removed and replaced	Survived	A
5/38/F	MICU (MD)	Group A streptococcal sepsis	Nov 16	Nov 16	ADT§	Died	A
6/38/M	Oncology (MD)	Head and neck tumor	Nov 20, Nov 26	Nov 20, Nov 23	ADT, implanted medication port removed and replaced	Survived	A
7/38/M	SICU (CA)	Temporal lobe hemorrhage	Oct 29	Oct 29	ADT	Survived	NA¶
8/59/M	SICU (CA)	Entero-cutaneous fistula; aorto-bifemoral bypass surgery	Nov 8	Nov 8	ADT	Survived	NA¶

*All infections (except in patient 1) developed after administration of intravenous fentanyl compounded at an out-of-state pharmacy; MD, Maryland; CA, California; NCCU, neurologic critical care unit; MICU, medical intensive care unit; SICU, surgical intensive care unit; NA, not applicable; ADT, antimicrobial drug therapy; MRSA, methicillin-resistant *Staphylococcus aureus;* PFGE, pulsed-field gel electrophoresis. †Intravenous fentanyl in 0.9% sodium chloride solution from a 250-mL (10 mcg/mL) bag prepared at compounding pharmacy A. ‡Strain A is the outbreak strain that was indistinguishable by PFGE from the fentanyl isolates. §The patient died of Group A streptococcal sepsis before the blood culture results for *S. paucimobilis* became available. ¶Isolates from patients 7 and 8 in the California hospital were not available for strain typing.

No breaches of infection control procedures or inappropriate blood culture practices were identified. Blood for culture was collected by staff in each unit rather than by a central vascular access team. No growth occurred from cultures of 50 noninoculated activated charcoal blood culture bottles; this finding was therefore not consistent with a pseudo-outbreak or intrinsic contamination of the bottles. Pharmacy records showed that 5 (83%) of 6 case-patients had received at least 1 intravenous dose of fentanyl (10 μg/mL in 250 mL 0.9% sodium chloride) within the 48 hours before *S*. *paucimobilis* bacteremia developed. No other common exposure was identified. We found that our hospital had outsourced preparation of 250-mL fentanyl bags to pharmacy A, which shipped several lots to the hospital every 2 weeks.

**Figure 2:**
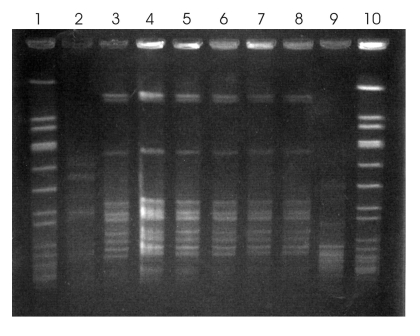
Results of pulsed-field gel electrophoresis (PFGE) of *Sphingomonas paucimobilis* isolates obtained in November 2007. Lanes 1 and 10, molecular weight marker; lanes 2–7, bloodstream isolates from patients 1–6, respectively; lane 8, isolate from contaminated fentanyl; lane 9, unrelated control isolate. Patients 2 through 6 received intravenous fentanyl within 48 hours before *S. paucimobilis* bacteremia developed and had isolates with a PFGE pattern indistinguishable from that of fentanyl isolates. Patient 1 did not receive intravenous fentanyl and had *S. paucimobilis* bacteremia with a distinct PFGE pattern.

Cultures of unopened samples from 1 implicated fentanyl lot grew *S. paucimobilis* that had a PFGE pattern indistinguishable from that of the isolates of the 5 patients who had received intravenous fentanyl (Figure 2). A sixth case-patient (patient 1; Table 2), who did not receive intravenous fentanyl, had *S. paucimobilis* with a unique PFGE pattern. Of 26 unopened bags from the implicated lot of fentanyl that were cultured, 16 (62%) grew *S. paucimobilis*. Cultures of 9 samples from 3 other fentanyl lots in use during the outbreak period produced no growth.

CDC’s multistate case-finding investigation determined that pharmacy A had distributed the contaminated fentanyl lot to 4 hospitals in different states. A California hospital that had received the implicated lot subsequently identified 2 additional case-patients who had *S. paucimobilis* bacteremia after administration of intravenous fentanyl from pharmacy A (Table 2). After diagnosis of bacteremia, these 2 case-patients received treatment with appropriate antimicrobial drugs without removal of existing central lines and were subsequently discharged from the hospital without complications from the bacteremia. At the California hospital, no unopened bags of the implicated fentanyl lot were available for culture, and the bloodstream isolates were not available for PFGE analysis. Specific lot numbers of fentanyl administered to patients were not available at either hospital. The 2 other hospitals that had received the implicated fentanyl lot did not detect any case-patients. The outbreak ended after the implicated fentanyl lot was removed from clinical areas. All bags of the implicated lot that could be located were tested for sterility at The Johns Hopkins Hospital and CDC before being discarded. The rest of the lot had either been used or was expired and destroyed. No product was recalled by pharmacy A.

Investigation found no evidence of tampering with or diversion of fentanyl within the Maryland hospital. We found stringent procedures in place to document and secure the narcotic chain of custody. No signs of tampering were visible on implicated bags of fentanyl that later grew *S. paucimobilis,* and the bags contained the expected concentration of fentanyl*.* We found that it was possible, although difficult, to remove and replace safety seals on the bags, which potentially could allow diversion of fentanyl without visible signs of tampering. Analysis of personnel access records from the automated medication and supply management system (Pyxis, Cardinal Health) did not identify any 1 person who had accessed >1 of the machines where the fentanyl was stored in the 4 hospital units with case-patients.

These 250-mL bags of intravenous fentanyl in 0.9% sodium chloride solution (10 μg/mL) are currently available only from pharmacy A and 1 other out-of-state compounding pharmacy. This preparation is the most frequently used at our institution and is not available from any pharmaceutical manufacturer. Because commercially available opioid alternatives at the desired concentration are not available, and to avoid disruption of patient care, our hospital continued to purchase the compounded preparation from pharmacy A. For 3 months after the outbreak, our microbiology laboratory performed sterility testing by culturing samples from each fentanyl lot received. None of these samples grew any bacterial or fungal pathogens. We do not have access to the results of FDA’s investigation into compounding practices at pharmacy A.

## Discussion

We describe a multistate outbreak of *S. paucimobilis* bacteremia that was associated with contaminated intravenous fentanyl prepared at an out-of-state compounding pharmacy. Astute observation by microbiology laboratory staff, good communication, and swift implementation of an epidemiologic investigation led to expeditious characterization of the source of the infections and termination of the outbreak. Pharmacy A prepares large quantities of compounded pharmaceutical preparations and distributes them to many states in anticipation of orders. In contrast to pharmaceutical manufacturers, traditional compounding pharmacies are not routinely inspected by FDA to ensure that they have the capacity to consistently produce high-quality drugs. In response to other infectious outbreak incidents, the FDA has warned compounding pharmacies that the high-volume production of pharmaceutical preparations in response to bulk orders constitutes manufacturing and is inconsistent with traditional pharmacy compounding (*17*,*18*). Current good manufacturing practice regulations, as defined by FDA, require end-product sterility testing, among other stringent controls, when sterile pharmaceutical products are manufactured (*19*). Although traditional pharmacy compounding fills a commercial void when FDA-approved, commercially available drugs cannot meet patients’ medical needs, the lack of end-product sterility testing and other standards of good manufacturing practice is a serious potential threat to patient safety when compounding pharmacies produce and distribute large quantities of sterile pharmaceutical preparations. Multistate distribution of compounded preparations makes outbreaks and clusters of infections even more difficult to detect and manage.

In 2001, as a result of a dispute over advertising restrictions, the Supreme Court ruled that the compounding provisions of the FDA Modernization Act of 1997 were unconstitutional (*20*–*22*). In 2002, the FDA issued guidance to clarify the FDA’s position on pharmaceutical compounding; the guidance identified factors that are considered in deciding whether to initiate enforcement action with respect to compounding (*1*). FDA historically has not taken enforcement actions against pharmacies engaged in traditional pharmacy compounding, but it has directed its enforcement against establishments whose activities are normally associated with a drug manufacturer. However, much of the investigation, regulation, and enforcement of compounding falls to state licensing boards (*2*,*18*). The American Society of Health-System Pharmacists (ASHP), the United States Pharmacopeia (USP), and the National Association of Boards of Pharmacy have issued practice and quality assurance guidelines for sterile compounding of pharmaceutical preparations (*23**,**24**)*. In 2004, USP chapter 797 put forth a set of enforceable standards for the compounding of sterile preparations (*25*); recently revised standards took effect on June 1, 2008 (*24*). These standards do not require end-product sterility testing for most compounded preparations (*24*). Some states require compounding pharmacies to comply with USP 797 and ASHP standards; however, a national survey found that many pharmacies are not fully compliant with ASHP quality assurance guidelines (*26*), and a 2006 survey documented incomplete awareness and implementation of USP 797 standards (*27*). From 1990 through 2002, the FDA received reports of >55 quality problems associated with compounded preparations (*28*); Table 1 shows reports of 6 such incidents published since 2002. An FDA survey in 2001 found 34% of tested preparations from compounding pharmacies failed to meet analytic testing standards, although none failed sterility tests (*28*).

Our investigation had limitations. Neither hospital could trace the lot numbers administered to each patient, so we could neither confirm which patients received contaminated lots of fentanyl nor assess an attack rate for receipt of the contaminated lots. We did not conduct a case–control study to calculate an odds ratio for administration of fentanyl being the primary risk factor for infection. Isolates from California case-patients were not available for strain typing. Finally, we do not have access to FDA’s investigational findings regarding pharmacy A, which might indicate the source of the contamination. Despite these limitations, the available evidence strongly suggests that contaminated fentanyl from pharmacy A was the source of this multistate outbreak of *S. paucimobilis* bloodstream infections.

FDA continues to face many serious and complex challenges (*29*,*30*). Our investigation, along with other similar published reports, indicates that the issue of large-scale pharmaceutical production and distribution by compounding pharmacies is also an urgent concern that requires attention. Pharmacies that compound and distribute large quantities of sterile pharmaceutical preparations without prescriptions for individual patients should be considered manufacturers and should be required to follow good manufacturing practices, including end-product sterility testing.

Until stricter regulations are imposed and enforced, hospital pharmacists and administrators must be cautious when outsourcing compounded pharmaceutical preparations and should consider the possibility of contaminated sterile pharmaceutical products when unusual organisms or patterns of disease are detected. Hospital personnel may be unaware that preparation of pharmaceutical products has been outsourced to a compounding pharmacy (*3*) and may not recognize the different regulatory requirements and potential implications of this decision. Healthcare facilities should strongly consider recording lot numbers of compounded medications administered to individual patients because this would aid investigations. Increased outsourcing of compounded pharmaceutical preparations makes surveillance for unexpected untoward events increasingly important. Compounding pharmacies should adhere to the standards set forth in USP 797 and should adopt more stringent quality control measures, including prerelease product testing, when preparing and distributing large quantities of sterile preparations.
